# Trabeculectomy Augmented with Limited Deep Sclerectomy and Cyclodialysis with Use of Scleral Tissue as a Spacer

**DOI:** 10.18502/jovr.v17i4.12342

**Published:** 2022-11-29

**Authors:** Tanuj Dada, Jyoti Shakrawal, Priyanka Ramesh, Anin Sethi

**Affiliations:** ^1^Glaucoma Research Facility & Clinical Services, Dr. Rajendra Prasad Centre for Ophthalmic Sciences, All India Institute of Medical Sciences, New Delhi, India; ^3^Tanuj Dada: https://orcid.org/0000-0002-3111-396X

**Keywords:** Refractory Glaucoma's, Cyclodialysis Augmented Trabeculectomy, Deep Sclerectomy.

## Abstract

Trabeculectomy remains the most commonly performed surgery for medically uncontrolled glaucoma. Its success in primary open angle glaucoma is approximately 82% in the initial year after surgery and 64% at the end of five years. Lower success rates have been found in secondary glaucomas like neovascular glucoma, uvietic glaucoma, post-traumatic glaucoma, and for repeat surgeries. To illustrate improvement of the efficacy of trabeculectomy, enhancement with cyclodialysis has been introduced. This involves the creation of a cyclodialysis cleft in a controlled manner to allow additional suprachoroidal drainage of the aqueous. Cyclodialysis is the result of the separation of the longitudinal ciliary muscle fibers from the scleral spur, which creates an additional pathway for aqueous humor drainage. However, such a cleft often closes on its own due to associated inflammation caused by the filtration surgery. Deep sclerectomy is a non-penetrating surgery that involves dissection of a scleral patch and excision of a block of scleral tissue, retaining a thin membrane for aqueous drainage. In this study, we introduce a novel surgical technique of combining trabeculectomy with a limited deep sclerectomy and a cyclodialysis in two pseudophakic patients who developed secondary glaucoma after vitreo-retinal surgery with silicone oil insertion. In this technique the excised scleral tissue obtained after deep sclerectomy was utilized as a spacer to maintain the patency of the cyclodialysis cleft.

##  INTRODUCTION 

### Case 1 

A 50-year-old female was referred with high intraocular pressure (IOP) after vitreoretinal (VR) surgery. The patient had a rhegmatogenous retinal detachment in the left eye (LE) for which she had undergone vitrectomy with silicone oil injection one year before and subsequently, after three months, underwent phacoemulsification with silicone oil removal. On examination, she had a visual acuity of 6/60 in the LE and 6/6 in the right eye (RE). LE IOP was 38 mmHg and 16 mmHg in RE. He was started on oral acetazolamide and maximal topical medications, which further reduced the IOP to 26 mmHg in LE. The patient was pseudophakic with extensive peripheral anterior synechiae on gonioscopy. The LE fundus showed a cup-disc-ratio of 0.8 with attached retina and an epimacular membrane.

### Case 2

A 38-year-old male presented with a history of LE VR surgery with silicone oil insertion for retinal detachment eight months back. The patient developed raised IOP after a few months. Silicone oil removal was done five months after the VR surgery. On examination, visual acuity was 6/24 in the LE and 6/6 in the RE. IOP in the LE was 44 mmHg while using maximum topical medication and 14 mmHg in the RE with no medications. On the slit-lamp examination, a few silicone oil droplets were present on the surface of the iris. Gonioscopy revealed emulsified silicone oil in the superior angle. The posterior segment showed an optic disc with a cup-disc-ratio of 0.9 in the LE. There was no significant cupping in examination of the RE fundus.

Since the IOP was not controlled by maximal medications and because of advanced glaucomatous optic neuropathy, we planned surgical management for both patients.

Considering the higher failure rate of conventional trabeculectomy with mitomycin-C (MMC) in post VR surgery glaucoma, presence of 360 ${}^{º}$ encircling band, and non-affordability precluding the use of glaucoma drainage devices, we planned to augment the conventional trabeculectomy surgery with deep sclerectomy and cyclodialysis.

##  SURGICAL TECHNIQUE

**Figure 1:**
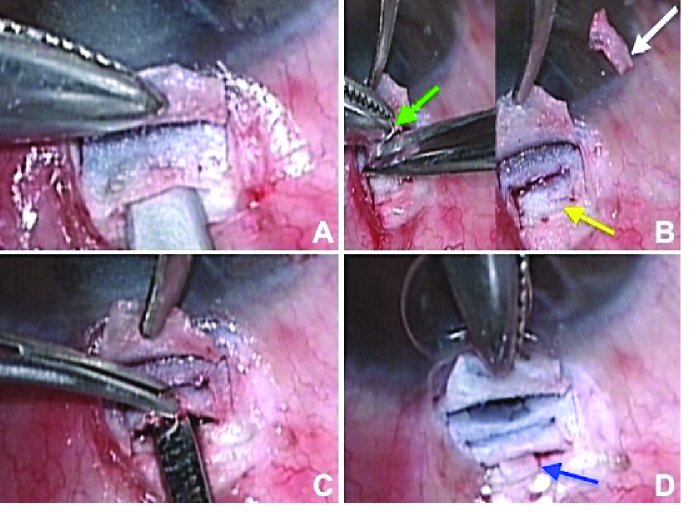
(A) Intraoperative image showing the creation of deep crater after making partial thickness scleral flap. (B) Dissection of the scleral block with Vanna's scissor (green arrow) and creating a deeper crater (yellow arrow), scleral block after dissection (white arrow). (C) Full-thickness incision made for cyclodialysis and cleft is created by using cyclodialysis spatula. (D) Scleral block is inserted in the cyclodialysis cleft (blue arrow).

The surgical procedure was performed under peribulbar anesthesia, with aseptic precautions, after receiving written informed consent from the patient. 0.01 ml of 0.01% MMC was injected in the sub-conjunctival space superiorly. A fornix-based conjunctival flap was dissected, hemostasis was achieved using bipolar electric cautery and the surgical site was thoroughly washed with the balanced salt solution. A partial-thickness scleral flap measuring 4 × 4 mm, with its base at the limbus, was dissected using a 2.3 mm crescent blade (Alcon, Fort Worth, USA). The remaining scleral bed was dissected further and a 3 × 3 mm block of scleral tissue was excised, thereby creating a deep sclerectomy [Figures 1A & 1B]. The excised block of scleral tissue was carefully preserved in balanced salt solution. Dissection of the deep scleral pocket was stopped at the scleral spur and no attempt was made to de-roof the canal of Schlemm. A full-thickness incision measuring around 2 mm was made 2 mm behind the scleral spur to expose the ciliary body. Using a cyclodialysis spatula, a controlled cyclodialysis was created [Figure 1C]. The trabeculectomy ostium measuring 2 × 1 mm was created at the base of the scleral flap and the silicone oil droplets from the anterior chamber were removed by using coaxial irrigation-aspiration. Retained silicone oil droplets in the anterior chamber even after silicone oil removal can go up in the superior angle and block trabecular meshwork. Therefore, this should be removed at the time of trabeculectomy, as retained droplets might block the trabeculectomy ostium. A peripheral iridectomy was made using Vanna's scissors. The preserved block of scleral tissue was rolled into a cylindrical configuration and inserted as a spacer in the cyclodialysis cleft [Figure 1D] followed by suturing it to the overlying sclera with 10–0 nylon monofilament suture (Aurolab Nylon sutures, double arm, Aurolab, India). The partial-thickness scleral flap was closed using two fixed 10-0 nylon sutures. Conjunctiva was closed with 8-0 Vicryl sutures (Ethicon; Johnson & Johnson, Aurangabad, India) at the limbus [Video 1].

Postoperatively, the patient was started on topical antibiotics, topical steroids, and cycloplegics. Patient was followed up on day one, week one, and at months one, two, and six, postoperatively. Postoperative anterior segment ocular coherence tomography (ASOCT) and ultrasound biomicroscopy (UBM) were performed.

##  RESULTS

**Figure 2:**
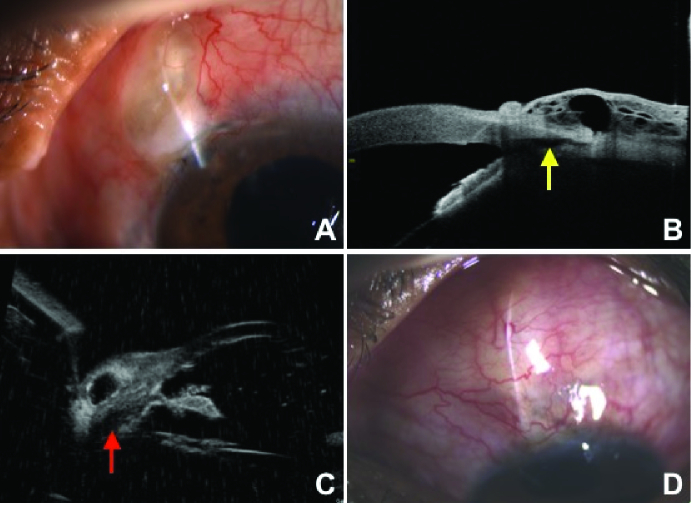
(A) Clinical photograph of case 1, six months postoperatively showing a diffuse, moderately elevated and mild vascular bleb. (B) ASOCT showing elevated cystic bleb with cyclodialysis cleft (yellow arrow). (C) UBM showing raised bleb with patent cyclodialysis cleft (red arrow). (D) Six months postoperative clinical picture of case 2 showing a diffuse, moderately elevated, and vascular bleb.

In case 1, on the first postoperative day, IOP in the LE was 10 mmHg with a diffuse, moderately elevated, and mildly vascular bleb with no leak (Height2 Extend2 Vascularity2 Seidel's0),^[4]^ with a formed anterior chamber. Visual acuity was maintained at 6/60. Six months after the surgery, IOP was 12 mmHg with a well-functioning bleb [Figure 2A]. ASOCT and UBM showed the patent cyclodialysis cleft with an elevated and microcystic bleb [Figures 2B and 2C].

In the second case, IOP dropped to 6 mmHg in the LE on day one postoperatively. Visual acuity improved to 6/18. There was a diffuse, moderately elevated, and moderately vascularized bleb with no leak (Height2 Extend2 Vascularity3 Seidel's0)^[4]^ [Figure 2D]. IOP was maintained at 10 mmHg, six months after the surgery.

##  DISCUSSION

Trabeculectomy is a full-thickness filtering procedure performed widely for glaucomas with uncontrolled IOP on maximum tolerable medical therapy. It has an efficacy of 75–100% in primary glaucomas.^[1,5]^ The main reasons for the failure of trabeculectomy include fibrosis in the sub-conjunctival space, scarring, and fibrosis of the Tenon's tissue, scleral fibrosis, and closure of the ostium.^[6]^ Trabeculectomy performed for secondary types of glaucoma such as neovascular glaucoma, post uveitic glaucoma, post penetrating keratoplasty glaucoma, aphakic glaucoma, and post silicone oil-induced glaucoma have a high risk of failure.^[5]^

Cyclodialysis combined with trabeculectomy was reported earlier to have achieved better outcomes. It involves the planned creation of a cyclodialysis cleft, creating an outflow tract to drain aqueous into the suprachoroidal space, along with the conventional outflow into the sub-conjunctival space and the Schlemm's canal.^[7]^ Sihota et al described ab-externo cyclodialysis along with trabeculectomy in post penetrating keratoplasty glaucoma with good outcomes.^[7]^ They performed the procedure in 45 eyes out of which 30 eyes had prior failed trabeculectomy surgery.^[8]^ Skalicky et al reported good long-term outcomes with no major complications in 55 eyes over a follow-up of 11.2 years.^[7]^ The cyclodialysis cleft created can undergo spontaneous closure with time, and to prevent this, many spacer materials such as Healon (Abbott Medical Optics, Abbott Laboratories Inc., Abbott Park, IL), high molecular weight hyaluronic acid, Ologen collagen matrix, Hydroxyethyl methacrylate capillary strip, and Teflon tube implants have been used.^[2]^

Deep sclerectomy was described initially as a nonpenetrating glaucoma surgery. It involves dissecting the scleral tissue retaining a thin membrane for aqueous to drain. The aqueous collects in the scleral lake and there is drainage through the intrascleral route. When combined with trabeculectomy, it allows for drainage via the sub-conjunctival as well as the intrascleral route. This also takes care of the extend of the bleb, thereby limiting the complications like overhanging blebs, thereby limiting the complications associated with a large bleb.^[3,9]^ Sharifipour et al also described a modified deep sclerectomy (MDS) technique which included exposure of ciliary body after deep sclerectomy to promote suprachoroidal flow. They found similar IOP reductions with MDS as of with trabeculectomy.^[10]^

In our technique, we have combined trabeculectomy with a limited deep sclerectomy and have used the excised scleral tissue block as a spacer to keep the cyclodialysis cleft open, preventing closure and fibrosis. The deep sclerectomy also creates a scleral lake to augment the drainage along with trabeculectomy and cyclodialysis. The use of patient's scleral tissue eliminates the need for synthetic materials that are more expensive and less available. Ologen implants have been previously used for this procedure,^[11]^ however, the implant is expensive and degrades after a few months. Our patients have shown promising results with no sight threatening complications during the short-term follow-up with the visualization of the cyclodialysis cleft as revealed on ASOCT and UBM. The utilization of deep sclerectomy tissue block as a stent to maintain the cyclodialysis cleft along with conventional trabeculectomy yields promising short-term results for IOP reduction in post vitrectomy glaucoma patients. This technique allows aqueous drainage via multiple pathways, thus increasing the efficacy of trabeculectomy. This unique technique might be a possible substitute for secondary glaucomas like neovascular glucoma, uvietic glaucoma, post-traumatic glaucoma, post VR surgery glaucoma, and for repeat surgeries. Further studies with a longer follow-up are required to look for its long-term success rate.

##  Financial Support and Sponsorship

The authors did not receive any financial support from any public or private sources.

##  Conflicts of Interest

The authors have no financial or proprietary interest in a product, method, or material described herein. No conflicting relationship exists for any author.
